# The Internet and science communication: blurring the boundaries

**DOI:** 10.3332/ecancer.2010.203

**Published:** 2010-12-14

**Authors:** R Warden

**Affiliations:** European Association for Cancer Research, EACR Secretariat, School of Pharmacy (PSB), University of Nottingham, Nottingham NG7 2RD, UK

## Abstract

Scientific research is heavily dependent on communication and collaboration. Research does not exist in a bubble; scientific work must be communicated in order to add it to the body of knowledge within a scientific community, so that its members may ‘stand on the shoulders of giants’ and benefit from all that has come before. The effectiveness of scientific communication is crucial to the pace of scientific progress: in all its forms it enables ideas to be formulated, results to be compared, and replications and improvements to be made. The sharing of science is a foundational aspect of the scientific method. This paper, part of the policy research within the FP7 EUROCANCERCOMS project, discusses how the Internet has changed communication by cancer researchers and how it has the potential to change it still more in the future. It will detail two broad types of communication: formal and informal, and how these are changing with the use of new web tools and technologies.

## Introduction

There has been much published about the role of the Internet in changing scholarly communication. There are few specific references to cancer researchers, and most of the studies are of a general nature. Cancer science, like every other academic discipline, has its accepted and well-known research methods and sources of information, such as peer-reviewed journals and known online databases of, for example, genomes or proteins. A 2010 article in the *International Handbook of Internet Research* comments on the phenomenon that ‘there is a high degree of mimetic professional organization and behaviour across the diverse cognitive domains of academic endeavour’ [3]. In other words, every academic discipline has its own learned societies, peer-reviewed journals, grant programmes and awards and prizes, and though the particulars may differ between fields, the general processes are the same. The same reasoning can be applied to different areas within cancer research: although in research methods and practices epidemiologists may differ from molecular biologists, the academic framework that surrounds them remains the same. In the absence of specific information on cancer researchers and their use of the Internet, I have used a ‘like-for-like’ approach with articles more general in subject.

Broadly speaking, it is possible to differentiate scholarly communication into two types: formal and informal. Formal communication is impersonal and takes the form of articles published in peer-reviewed journals, and to a lesser degree the presentation of results at meetings in the form of talks, abstracts and posters. It is expected to be a robust and reliable piece of information, reflecting its peer-reviewed, completed status [1]. Informal communication, on the other hand, is traditionally between partners who know each other and wish to exchange anything from ideas and results to draft papers and preprints. The development of Internet technologies, as I will discuss later, has changed the nature of informal communication and widened its potential to facilitate learning and collaboration. It has been argued that the Internet and electronic publishing has begun to blur the line between formal and informal communication, and alter the traditional roles occupied by the producers, processors, and users of information [21]. Self-publishing of a completed research report on an institute or personal website, including semi-formalized ones, such as academia.edu (see http://www.academia.edu) is one example of this: the publication does not fall into the traditional model of a journal article, yet it is clearly not an informal communication either.

## Formal, peer-reviewed communication

Scientific journals are clearly of huge importance to cancer scientists. Scientific research can be described in terms of a cycle consisting of idea discovery, gaining funding/approval, conducting the research, and disseminating the results. The cycle begins in the consultation of existing publications, and ends, ideally, in the publication of results, which then go on to influence further research. The increased use of electronic journals, compared with print journals, has been well documented. There is some evidence that frequent Internet use for information retrieval and communication is associated with the increase in publication production by scientists [1,30]. A Finnish study [30] surveyed academics from a wide range of disciplines and found that scholars perceived that electronic resources had made it significantly easier to identify, access and locate material, and also extended the range of literature at their disposal. They also reported less frequent use of physical libraries and less time spent browsing for information. To a lesser extent, the surveyed academics reported that using electronic resources had inspired new ideas and improved the quality of their work.

## Beyond the electronic journal

In 2002, Andrew Odlyzko reported that electronic journals were being read about as often as their printed journal counterparts and predicted that paper journals would soon be eclipsed by electronic ones, and print would eventually become irrelevant. Nentwich [13] argues that the Internet has radically changed the scholarly publication system. His examples, as well as electronic journals, include digital ‘working paper’ archives that give access to research literature at an early stage, research libraries (‘cybraries’) offering access to digital repositories of papers, and new forms of scholarly publications that would not have been possible in the traditional paper environment and can only be produced in digital formats. Some of these new formats include hypertexts, which present knowledge differently; multimedia, which uses new ways to convey messages to the reader; and the new practice of communicating research results via databases. Nentwich argues that the entire process of formal communication is fundamentally changing with the development of new web technology.

Yet Odlyzko [15], as well as predicting the rise of the electronic journal, also comments on the inertia of journal publishers and their slowness in changing their publishing methods along with their medium. Although agreeing that articles will become more accessible because ‘the realization will spread that anything not easily available on the Web will be almost invisible’ (p18), he also predicts that the traditional format of peer-reviewed text-based articles will remain the prevalent method of communicating results for some years to come. Yet there is evidence that scientific journals have the potential to adapt and change: a small handful of journals have opened up online interactive discussion, including the BMJ Website, which allows readers to post ‘rapid response’ comments on published articles. Many journals have also embraced the online tools that are available to them to maximize their usefulness and interest. Journals such as *Nature* and *Science* include features on their websites, such as blogs, RSS feeds, podcasts and videos, and *Science* maintains a presence on social network sites Facebook and Twitter.

The Web has allowed data to be represented and analysed in new ways that greatly enhance its value and the potential to extract useful findings by allowing it to be integrated and compared with other data. One approach to such integration is through the annotation of different bodies of data using common controlled vocabularies or ‘ontologies’. According to Renear and Palmer [18], the use of ontologies is particularly key in the biological sciences, in order to identify what is biologically and clinically significant in the swathes of data being generated. One such endeavour is the Gene Ontology (GO) project, which aims to standardize ‘the representation of gene and gene product attributes across species and databases’ (see http://www.geneontology.org/). Although many biological ontologies were originally developed independently, the need for interoperability has driven collaboration, a good example being the Open Biomedical Ontologies (OBO) (see http://www.obofoundry.org/), which has participating projects that include Microarray Gene Expression Data (MGED) and BioPAX, for biological pathways data. Ontologies are widely used: according to Rhee *et al* [19] at the time of writing, there were 2960 citations for the Gene Ontology project in version 3.0 of the ISI Web of Knowledge.

## A surfeit of information

A 2008 study comparing scholarly e-reading patterns in Finland, the United States and Australia found that use of search engines is overwhelmingly the most popular method for finding electronic articles, followed by browsing, citations and colleagues [28]. One problem for researchers looking for information online is the plethora of information available to them through searching. Publishers and online repositories offer their own search tools, but the sheer number of these creates its own problem—how to find the right place to search. Google Scholar was introduced in 2004 as a simple search interface to locate scholarly articles. The many studies and reviews of this tool are summarized in Jacsó [9], who ultimately judges it to be a useful yet flawed source of information. One study by Neuhaus *et al* [14] found a marked discrepancy between Google Scholar’s coverage of open access journal databases and all other databases investigated (i.e. fee-based restricted databases). According to their results, the mean score for coverage of open access journal databases was 95% and the mean score for all other databases was 57%.

Search habits may also be a problem when trying to find useful articles. A 2006 study [10] reported that for the period recorded, 81% of Google searchers viewed only one results page. Although this research was conducted using a random sample of the general population, it seems likely that this will hold true for least some researchers who use Google and Google Scholar to find information. Those who only view one page of results for any search are likely to miss important results, and their ability to find information will rely solely on a search engine’s algorithms.

The number of articles per year read by university science faculty members has steadily increased, while the amount of time spent on each article has steadily decreased [28]. Renear and Palmer [18] put this down to increasingly sophisticated strategic reading, using indexing and citations as indicators of relevance and abstracts and literature reviews as surrogates for full papers. As the online environment has enabled indexing, recommending, and navigation to become more sophisticated, these strategic reading practices have intensified. Many tools have been designed specifically to query the databases of biomedical publications such as PubMed, using sophisticated ontologies to avoid such issues as ambiguity and variation of search terms in order to return the most relevant results. Some examples, summarized in Spasic *et al* [26], include UMLS (Unified Medical Language System) (see http://www.nlm.nih.gov/research/umls) and Textpresso (see http://www.textpresso.org/), an information retrieval system operating at the sentence level. These tools enable researchers to optimize their strategic reading practices and maximize the potential of their information searches.

## Informal communication

Informal communication is as much a part of a cancer scientist’s career as the publication of articles. Email has been a prevalent method of communication for years, and it has been shown that scientists who collaborate use email more frequently than those who do not collaborate [32]. Walsh *et al* argue that Internet communication does not necessarily lead to collaboration, but facilitates it, especially collaboration over a great physical distance. This ties in with the concept of the ‘invisible college’, where scientists in a certain field of expertise form an informal network of communication in which to share ideas. The development of new Web 2.0 tools, as described below, provides still further opportunity for collaboration and discussion.

The way in which scientists communicate informally is changing as the Web develops. This is defined by the transition from ‘Web 1.0’ to ‘Web 2.0’. Web 1.0 is generally used to describe the ‘old’ system of passively accessing static information from the Web. Web 2.0 is an umbrella term for a growing range of Internet tools and technologies that are typified by being interactive and collaborative and allowing information sharing and user-generated content.

As the Web brings informal communication into the public domain, it is more important to differentiate between the different levels within that communication. While communications, such as preprints, draft papers and quality-controlled blog posts, occupy the higher end of the scale on authority and trustworthiness, the universal access provided by the Web jumbles these together with everything else: casual chats in a forum, blog or social media platform, unverified data and preliminary ideas and theories. This makes it more important than ever for both scientists and the public to have access to trusted platforms that filter the swathes of information to include only that, which will be useful to them. It is also very important for the researchers of the future to develop an awareness of the issues surrounding data trustworthiness on the Web and not rely, as I will mention later, solely on strongly branded search engines as their only portal to information.

What many Web 2.0 technologies recognize is that scientific knowledge is not just made up of data and results. Giordano [7] identifies two types of scientific information produced by every lab: public results that can be scrutinized and replicated, and ‘private research products’ that can include methods, bench techniques, workflows, software and algorithms. In general, these private methods are not publishable, but they are the driving force behind producing the data and results that are published. If it is important to share results, then it is equally important, yet much more difficult, to share the methods behind these results. A scientific journal article could be described as only a ‘snapshot’ of a given problem and solution. Because the Web provides virtually unlimited space, it can allow scientists to publish their lab notebooks and different methods attempted as well as the data that was finally obtained, meaning readers can understand not only the results but also the exploratory processes that led to these results.

Blogging is one Web 2.0 tool that is well suited to informal communication by researchers. The readership that can be reached by blogging far surpasses that of any form of informal communication that has gone before: it can be used to communicate directly with the public and popularize science as well as to communicate and discuss ideas with other scientists. Batts *et al* [2] argue that though scientific developments are made in individual labs, science as a whole is furthered by ‘a series of ongoing conversations, from a Nobel Prize winner’s acceptance speech to collegial chats at a pub’. Blogging about science takes these conversations from the private into the public sphere and allows other scientists to become involved in a level of discussion and debate that could never be achieved in most scientific journals. Allowing the public to witness such debates by holding them in a public forum such as blogs can only increase public knowledge of the complexity and importance of basic cancer research.

The popular news media are frequently considered to be poor at reporting accurately on basic cancer research: notably they have been accused of a tendency to sensationalize items, report basic developments as though they are preventative or clinical breakthroughs, disregard previous, conflicting studies in favour of a ‘new angle’ and include no caveats to account for scientific doubt or uncertainty [4,22,11]. The exceptions to this are popular science magazines: publications such as *New Scientist* and *Scientific American* that deliver scientific news via articles and features like a newspaper, but cite the sources of information like a journal [12]. Blogging can enable scientists to directly engage the public in ‘good science’, focusing on their own area of expertise with an authority and depth that cannot be achieved by most newspaper or magazine articles.

An important positive result of reading and writing blogs is that it can foster an interest in ideas and applications that are outside a researcher’s particular area of specialty. In 2007, *New Scientist* asked various leading cancer researchers what was required to get ahead in cancer research [24]. A recurring point these experts mentioned was the need for the researcher to have an understanding of the wider context of their work, which may lead to collaborations and clinical applications that might otherwise never occur to them.

One key issue with Web 2.0 technology, as I will discuss later, is the presence of doubt over the provenance or accuracy of information that is posted. There are various methods for filtering the many blogs that are available, for example ‘blog awards’ such as the Research Blogging Award (see http://researchblogging.org/static/index/page/awards), and communities of pre-selected blogs such as ScienceBlogs.com (see http://scienceblogs.com/) and ResearchBlogging.org (see http://researchblogging.org/). ResearchBlogging.org is a good example of self-regulation by a Web 2.0 community. The site automatically aggregates only blog posts about peer-reviewed research from a list of pre-approved blogs, using a piece of code inserted into relevant posts by the author. If a post does not fit into the site’s guidelines, it can be reported and discussed by the member bloggers and removed if necessary.

The use of Web 2.0 tools by cancer scientists is still in its infancy and may be hampered by fears over (a) accuracy of user generated content and (b) confidentiality of results.

## Accuracy of Web 2.0

Rowlands *et al* [20] comment on the growing tendency among scholarly information seekers to look solely for ‘the answer’ rather than information in a particular format, such as a journal article. They also comment on the tendency, especially in the younger generation of Internet users who have grown up using the Web for all their information-seeking needs, to trust and use strongly branded and familiar search engines, such as Google, over any other source. One conclusion to be drawn from this is that researchers may be tempted to turn to perhaps the most familiar source of collaborative information on the Web, which is often highly ranked in search engine results: Wikipedia. In 2005, *Nature* surveyed more than 1000 *Nature* authors and found that more than 70% had heard of Wikipedia and 17% of those consulted it on a weekly basis. As part of the same study, *Nature* selected 50 Wikipedia articles on subjects that represented a broad range of scientific disciplines and had them peer-reviewed. On average, the articles contained four errors each and some reviewers reported that the articles were poorly structured and confusing [6]. However, it is also worth noting that the study compared Wikipedia against a respected encyclopaedia: Encyclopaedia Britannica. For the average four errors per article in Wikipedia, Britannica contained an average of three. The perceived inaccuracies in both sources may be the result of submitting articles from lay encyclopaedias to renowned experts on the topic in question, who have a level and depth of knowledge that most general contributors do not possess.

As these new collaborative technologies develop, science can formulate its own tools based on similar concepts to that of Wikipedia, only of a more specialized nature. One example of how Web 2.0 technologies can advance biological research is *OpenWetWare* (see http://openwetware.org/wiki/Main_Page). OpenWetWare is a specialized wiki on which researchers can share lab protocols, data and ideas in biological science and engineering. The developers aim to avoid the accuracy problems of Wikipedia by ensuring that users can only make changes after they have registered and demonstrated that they belong to a legitimate research organization. There are many scientific wikis being developed in the same vein, such as WikiPathways (see http://www.wikipathways.org/), a collaboratively curated database of biological pathways, and WikiGenes (see http://www.wikigenes.org/), a wiki that acts as a portal to databases and articles on genes, proteins and chemical compounds. Scientific wikis tend to have higher barriers to editing than Wikipedia, and stricter tracking of authorship and changes made to articles. WikiGenes offers strong authorship attribution, with every change able to be tracked back to its originator and users able to rate each other based on the quality of their contributions. This allows self-regulation based on users’ desire to maintain a good reputation on the site (Hoffmann, 2008). This self-regulation can be applied to any Web 2.0 technology: if a strong online community is formed, based on people’s real identities, the desire to maintain a good reputation online could be just as strong as the desire to do so in real life.

Hoffmann (2008) describes scientific wikis as ‘dynamic publications’, compared to the ‘static’ traditional methods of scientific publication, where a journal article has a set number of authors and a precise date of publication (the ‘snapshot’ described earlier). This inflexibility can especially become an issue with centrally controlled and curated databases: the larger the database, the more there is for the curators to do and the sooner it may become out of date as new discoveries are made. The level of expertise required to curate a database of biological data may often be very specific, and no small team of curators can hope to achieve specific expertise in every aspect that their database covers. Since they could have an almost unlimited number of authors and be constantly updated, scientific wikis could potentially always be up to date. Hoffmann posits that as a result a scientific wiki would contain no ‘explicit errata’, only improved versions of an article.

However, the concept of virtually unlimited authors is currently theoretical, since only limited numbers of scientists currently register on and get involved in science wikis. In a 2008 letter to *Science* the developers of several online collaborative tools [8] denied that individual curators or editors can match the collective knowledge of the scientific community. Instead they bemoaned that ‘so far, the challenge is not chaos but lack of participation’. If the renowned experts that acted as peer-reviewers in the *Nature* study on Wikipedia actively contributed their knowledge to the wiki, the quality of the encyclopaedia would be greatly increased. The same applies to specialist scientific wikis: the more active users there are reading, commenting on and editing entries, the more eyes there will be to spot errors or areas for improvement, and the more brains will be at work contributing ideas and information.

## Confidentiality

The open, collaborative nature of some Web 2.0 technologies and the idea of publishing raw data as well as the methods and techniques behind that data goes against certain principles of academic culture that have traditionally been held to be important, namely competitiveness and the confidentiality of results. Researchers are constantly in pursuit of *new* knowledge: the traditional publishing model recognizes the scientist who has published first as the originator of that knowledge. As Giordano [7] puts it, ‘if you do not publish research findings first, you, in effect, have not published at all’. The concept of the ‘selfish scientist’ [29,7], needing to protect their ideas and discoveries in order to publish first and thereby retain funding and further their career, goes against the basic precepts behind many Web 2.0 technologies. As the tools are developed to allow sharing and collaboration on an unprecedented scale, an academic culture that rewards secrecy and self-interest may become more out of place.

A 2009 study [23] appears to show unwillingness by some researchers to hand over raw data even after publication. The study found that of ten sets of authors publishing papers in either *PLoS* (Public Library of Science) *Medicine* or *PLoS Clinical Trials*, only one shared their raw data when requested to do so, despite the editorial policy of *PLoS* that authors share their data with other investigators. A 2001 study [17] found the same result from authors appearing in the British Medical Journal: only one author out of the 29 approached shared their data. Many factors may explain why data sharing, even after publication, is not always forthcoming: preparing data for others requires some work, there can be confidentiality issues, there may be a competitive edge to be gained by having data others do not, and there may even be a fear of having one’s findings debunked or contradicted. Funding bodies often include obligations regarding retention of and providing access to research data in the grant agreement or contract through which funding is provided. However, there is evidence that sharing detailed research data after publication can be beneficial to authors by increasing their citation rate [16]. It is also clearly beneficial to the development of science as a whole as data can be tested, replicated and improved upon.

## Conclusion

When Tim Berners-Lee invented the World Wide Web in 1989, he envisaged it as a collaborative workspace for his fellow scientists at the CERN institute to share ideas across a network. Years later, the Web appears to be returning towards Berners-Lee’s original vision, with web users more and more willing to contribute actively to the content they see online. With access to the Internet becoming more widespread, from ultrafast broadband connections and increasing mobile wireless access to the rapid rise in computer access in developing countries, the Web’s potential is growing. As the Web develops, communication by cancer scientists, both formal and informal, is in a process of transformation. Use of the Internet and email is prevalent over other communication methods, and studies have shown that more frequent use of these is linked to increased collaboration and productivity by researchers. Information seeking has changed: with the increased amount of information that is available through the Internet, researchers are adjusting their methods in order to identify and filter out what is useful. Although the Internet creates the problem of a surfeit of information, it also offers the solution: the development of new online tools to navigate the Web and interpret complex data in increasingly sophisticated ways. Another key area of potential growth is Web 2.0 technology: collaborative projects that allow researchers to share ideas and expertise and even collaboratively analyse data online. If such projects are to reach their full potential, a change is required in the general attitude of the scientific community: from viewing the Web as a source of passively acquired information to viewing it as a platform for sharing and collaboration.

## References

[1] Barjak F (2006). The role of the Internet in informal scholarly communication. Journal of the American Society for Information Science and Technology.

[2] Batts SA (2008). Advancing Science through Conversations: Bridging the Gap between Blogs and the Academy. PLoS Biology.

[3] den Besten M (2010). Research in e-Science and Open Access to Data and Information. The International Handbook of Internet Research.

[4] Dentzer S (2009). Communicating Medical News—Pitfalls of Health Care Journalism. New England Journal of Medicine.

[5] Gene Ontology Consortium (2006). The Gene Ontology (GO) project in 2006. Nucleic Acids Research.

[6] Giles J (2005). Internet encyclopaedias go head to head. Nature.

[7] Giordano R (2007). The Scientist: Secretive, Selfish or Reticent? A Social Network Analysis. E-Social Science conference.

[8] Hu JC The Emerging World of Wikis. Science.

[9] Jacsó P (2008). Google Scholar revisited. Online Information Review.

[10] Jansen BJ, Spink A (2006). How are we searching the World Wide Web? A comparison of nine search engine transaction logs. Information Processing & Management.

[11] Jensen JD (2008). Scientific Uncertainty in News Coverage of Cancer Research: Effects of Hedging on Scientists’ and Journalists’ Credibility. Human Communication Research.

[12] Lewison G, Turnbull T (2010). News in brief and features in New Scientist magazine and the biomedical research papers that they cite, August 2008 to July 2009. Scientometrics.

[13] Nentwich M (2005). Quality control in academic publishing: challenges in the age of cyberscience. Poiesis & Praxis: International Journal of Technology Assessment and Ethics of Science.

[14] Neuhaus C (2006). The Depth and Breadth of Google Scholar: An Empirical Study. Libraries and the Academy.

[15] Odlyzko A (2002). The rapid evolution of scholarly communication. Learned Publishing.

[16] Piwowar HA, Day RS, Fridsma DB (2007). Sharing Detailed Research Data Is Associated with Increased Citation Rate. PLoS ONE.

[17] Reidpath DD, Allotey PA (2001). Data Sharing in Medical Research: An Empirical Investigation. Bioethics.

[18] Renear AH, Palmer CL (2009). Strategic Reading, Ontologies, and the Future of Scientific Publishing. Science.

[19] Rhee S (2008). Use and misuse of the gene ontology annotations. Nature Reviews Genetics.

[20] Rowlands I (2008). The Google generation: the information behaviour of the researcher of the future. Aslib Proceedings: New Information Perspectives.

[21] Russell JM (2002). Scientific communication at the beginning of the twenty-first century. International Social Science Journal.

[22] Russell C (1999). Living can be hazardous to your health: How the news media cover cancer risks. Journal of the National Cancer Institute Monographs.

[23] Savage CJ, Vickers AJ (2009). Empirical Study of Data Sharing by Authors Publishing in PLoS Journals. PloS one.

[24] Smeaton Z (2007). How to get ahead in a cancer research career. New Scientist.

[25] Smith B (2007). The OBO Foundry: coordinated evolution of ontologies to support biomedical data integration. Nature Biotechnology.

[26] Spasic I (2005). Text mining and ontologies in biomedicine: Making sense of raw text. Briefings in Bioinformatics.

[27] Tenopir C (2009). Electronic journals and changes in scholarly article seeking and reading patterns. Aslib Proceedings.

[28] Tenopir C Scholarly E-reading Patterns in Australia, Finland, and the United States: A Cross Country Comparison. http://www.ifla.org/IV/ifla74/papers/160-Tenopir_Wilson_Vakkari_Talja_King-en.pdf.

[29] Thomson H (2006). ‘Me-Science’: the New e-Science. GRIDtoday.

[30] Vakkari P (2008). Perceived influence of the use of electronic information resources on scholarly work and publication productivity. Journal of the American Society for Information Science and Technology.

[31] Waldrop MM (2008). Science 2.0 - Is Open Access Science the Future?. Scientific American.

[32] Walsh JP (2000). Connecting minds: Computer-mediated communication and scientific work. Journal of the American Society for Information Science.

